# Molecular and Drug Resistance Characteristics of *Haemophilus influenzae* Carried by Pediatric Patients with Adenoid Hypertrophy

**DOI:** 10.3390/microorganisms13081764

**Published:** 2025-07-29

**Authors:** Nan Xiao, Jia-Hao Qin, Xiu-Ying Zhao, Lin Liu

**Affiliations:** 1 Department of Laboratory Medicine, Beijing Tsinghua Changgung Hospital, School of Clinical Medicine, Tsinghua University, Beijing 102218, China; xna01053@btch.edu.cn; 2 Dalian University Collage of Medicine, Dalian 116622, China; qin-jiahao@foxmail.com

**Keywords:** *H. influenzae*, adenoid hypertrophy (AH), Clonal Complex 11, β-lactamase-positive ampicillin-resistant (BLPAR), imipenem-resistant

## Abstract

Purpose: The adenoid microbiota plays a key role in adenoid hypertrophy (AH). This study explored the molecular epidemiology and antimicrobial resistance of *Haemophilus. Influenzae* (*H. influenzae*) strains in pediatric AH patients. Methods: Retrospective analysis of pediatric AH patients undergoing endoscopic adenoidectomy. Adenoid tissue samples were cultured to screen for pathogens. *H. influenzae* strains were identified by 16S rRNA sequencing and serotyped via q-PCR. Multilocus sequence typing (MLST) and ftsI gene analysis were conducted using PubMLST. β-lactamase genes (*bla*_TEM-1_, *bla*_ROB-1_) were detected by PCR, and antibiotic susceptibility testing (AST) was performed using the Etest method. For imipenem-resistant strains, the acrRAB efflux pump gene cluster and ompP2 porin gene were sequenced and compared with those of the wild-type strain Rd KW20. Results: Over 8 months, 56 non-duplicate *H. influenzae* strains were isolated from 386 patients. The detection rate was highest in children under 5 years (30.5%) compared to those aged 5–10 years (13.4%) and 10–15 years (8.7%). Of 49 sub-cultured strains, all were non-typeable *H. influenzae* (NTHi). MLST identified 22 sequence types (STs) and 13 clonal complexes (CCs), with CC11 (26.5%), CC3 (14.3%), and CC107 (14.3%) being predominant. Common STs included ST103 (22.4%), ST57 (10.2%), and ST107 (10.2%). Most strains belonged to the ftsI group III-like+ (57.1%). β-lactamase positivity was 98.0% (48/49), with *bla*_TEM-1_ (95.9%) and *bla*_ROB-1_ (18.4%) detected. AST showed low susceptibility to ampicillin (10.2%), amoxicillin–clavulanate (34.7%), azithromycin (12.2%), and trimethoprim–sulfamethoxazole (14.3%). Among the β-lactamase-positive strains, 44/48 were β-lactamase-positive ampicillin-resistant (BLPAR); none were β-lactamase-negative ampicillin-resistant (BLNAR). Imipenem susceptibility was 91.8% (45/49). No carbapenemases were found in the imipenem-resistant strains, but mutations in acrRAB (88.12–94.94% identity) and ompP2 (77.10–82.94% identity) were observed. Conclusions: BLPAR NTHi strains of CC11 are major epidemic strains in pediatric AH. Imipenem resistance in *H. influenzae* likely results from porin mutations rather than carbapenemase activity. Enhanced surveillance of *H. influenzae*’s role in AH and its resistance patterns is warranted.

## 1. Introduction

Adenoid hypertrophy (AH), marked by the enlargement of the adenoids, is a prevalent cause of upper respiratory obstruction in pediatric patients. This condition often predisposes children to recurrent infections of the upper respiratory tracts, owing to airflow obstruction and disruption of immune mechanisms [1]. Human adenoids and palatine tonsils, with their numerous folds and crypts, serve as habitats for commensal bacteria of the upper respiratory track. Consequently, the adenoid microbiota plays a pivotal role in the development of various diseases of the upper airways, such as otitis media, adenotonsillitis, rhinosinusitis, and AH [2]. The pathogen reservoir hypothesis posits that the adenoids act as a reservoir of conditional pathogenic bacteria, potentially leading to diseases [2,3]. The most prevalent and abundant adenoid microbiota could be classified into several core genera, including *Gemella*, *Haemophilus*, *Streptococcus*, *Neisseria*, *Porphyromonas*, and *Fusobacterium* [3,4,5]. Notably, differences in the tonsil crypt microbiota between children and adults have been observed, with opportunistic pathogens including *Haemophilus influenzae (H. influenzae)*, *Neisseria* sp., and *Streptococcus pneumoniae* being almost exclusively detected in young children [2,5]. It is evident that *H. influenzae* is one of the most frequently detected opportunistic pathogens in the adenoid microbiota [3,4,5]. This small, fastidious Gram-negative coccobacillus typically causes respiratory tract infections and invasive diseases [6,7] and is known to diffusely infiltrate the tissue of adenoids [5].

The resistance of *H. influenzae* to β-lactam antibiotics can have important clinical implications especially when it causes infections among children. Two important mechanisms are frequently studied regarding the resistance of *H. influenzae* to aminopenicillins. One involves beta-lactamases, and the other mutations of penicillin-binding proteins (PBPs), especially PBP3, due to *ftsI* mutations [8,9]. The most frequent β-lactamases identified in *H. influenzae* are of the *bla*_TEM-1_ type and *bla*_ROB-1_ type [10]. Other kinds of β-lactamases, including *bla*_ROB-11_ and *bla*_TEM-15_, were seldomly reported [11] or less effective [12] or confer significant competitive disadvantages for *H. influenzae* [13]. Besides the resistance mediated by the production of β-lactamases, another mechanism entails the decreased affinity of AMP for penicillin-binding proteins (PBPs) involved in peptidoglycan synthesis, especially PBP3 encoded by the gene *ftsI* [14,15]. Epidemiological evidence shows an increase in the spread of non-β-lactamase-dependent resistance to β-lactam antibiotics. Specific amino acid substitutions in the *ftsI* gene, such as in the KTG (Lys-Thr-Gly) and SSN (Ser-Ser-Asn) motifs in PBP3, alter the protein’s structure, leading to resistance to β-lactams. While an initial variation in PBP3, either R517H or N526K, leads to low-level ampicillin resistance, its combination with the S385T substitution results in high-level resistance patterns. When the mutations L389F and S385T were added to R517H and N526K, the cephalosporin minimum inhibitory concentrations (MICs) could increase [16]. It was reported that during the early 2010s the ampicillin resistance rate could be as high as 70% among *H. influenzae* isolates from young children in Korea and Japan [7], while in Brazil, the resistance rate in the same period was only 17.1% [17]. Conversely, the resistance rate to third-generation cephalosporins, such as ceftriaxone and cefotaxime, was still relatively low [7,10].

The polysaccharide capsule of *H. influenzae* was found to be the main toxicity factor causing serious infections, including meningitis and pneumonia, especially in children aged <5 years [18,19,20]. However, with the widespread implementation of a vaccine against the capsule [21], the incidence of diseases caused by encapsuled *H. influenzae* has declined [20]. Already 10 years ago, people recognized the increasing importance of non-typeable *H. influenzae* (NTHi), which lacks a polysaccharide capsule, as a pathogen in upper and lower respiratory tract infections and invasive diseases [7,20,22,23].

So far, there has been limited information on the molecular epidemiology and antimicrobial resistance of *H. influenzae* isolated from pediatric patients with AH in Northern China. The recently published relevant literature only reports data on *H. influenzae* causing invasive diseases. However, due to the complex composition of the study populations, the conclusions drawn lack strong specificity. In this study, we retrospectively investigated the *H. influenzae* strains cultured from nasopharyngeal tissue samples, which were routinely collected during the operations of endoscopic adenoidectomy in patients with AH. Due to restrictions on pediatric medication, β-lactam antibiotics are the first-line drugs when systemic therapy is indicated [14]. Therefore, we focused our research on the resistance mechanisms of β-lactams. We analyzed by MLST mutations of the *ftsI* gene and the genes involved in β-lactamase and antibiotic resistance in *H. influenzae* strains. The potential mechanisms of drug resistance were also analyzed.

## 2. Material and Methods

### 2.1. Data Collection

Pediatric patients suffering from AH underwent endoscopic adenoidectomy in the Department of Otolaryngology at a tertiary hospital in Beijing, China, from January 2024 to August 2024. All data were retrospectively analyzed including information regarding diagnosis, gender, age, and the results of routine bacterial culture. The analysis was conducted anonymously and complied with the requirement of the ethical approval issued by Ethics Committee of Beijing Tsinghua Changgung Hospital.

### 2.2. H. influenzae Isolation

The nasopharyngeal tissue samples were routinely sent to the microbiological laboratory for culture after endoscopic adenoidectomy in order to screen for opportunistic pathogens. All tissues were cultured on chocolate agar and Columbia blood agar (OXOID Ltd., Thermo Fisher Scientific, Hampshire, UK) at 37 °C with 5% CO_2_ for 18–24 h. All the suspicious greyish round-shaped colonies only growing on chocolate agar were identified using matrix-assisted laser desorption ionization–time of flight mass spectrometry (MALDI-TOF MS) (Bruker Corporation, Bremen, Germany). If the *H. influenzae* strains were successfully sub-cultured, they were numbered in sequence using the numbering system of the strain bank in our laboratory and stored properly for further analysis.

### 2.3. Typing of the H. influenzae Strains by q-PCR Method

The capsule gene *bexA* was tested by PCR with the primers reported by Yuan et al. [10]; the primers of the capsule gene *bexB* were provided by Davis GS et al. [24]. In order to identify all the *bexA*-positive and/or *bexB*-positive strains, we performed a quantitative PCR test according to the protocol from the WHO manual [25]. DNA isolation was performed using a commercial silica membrane-based spin column kit (EasyPure^®^ Bacteria Genomic DNA Kit, TransGen Biotech, Beijing, China), following the manufacturer’s protocol for the efficient purification of high-quality genomic DNA. qPCR analysis was conducted using a QuantStudio 5 Real-Time PCR System (Applied Biosystems™, Thermo Fisher Scientific, Foster City, CA, USA), equipped with an advanced optical detector for precise fluorescence quantification. Negative controls were included in every run to monitor contamination or non-specific amplification, while the gene *hdp* was used as a positive control to validate assay sensitivity and amplification efficiency. Primers and probes were synthesized by Beijing Ruibo Xingke Biotechnology Co., Beijing, China. The primers and probes used are listed in the Supplementary Materials. The reactions utilized Taq polymerase (TaKaRa Ex Taq™ HS, Takara Bio, Kusatsu, Japan).

### 2.4. Identification of H. influenzae

All numbered *H. influenzae* strains were re-identified using 16S rRNA sequences with the universal primers 27f and 1492r [26]. All the 16S rRNA sequences were analyzed by the online platform EZbiocloud (http://www.ezbiocloud.net/ accessed on 13 October 2024).

### 2.5. MLST Analysis and Minimum Spanning Tree Analysis

The seven gene loci used in multilocus sequence typing (MLST) were *adk*, *atpG*, *frdB*, *fucK*, *mdh*, *pgi*, and *recA* [27]. Sequencing of the seven loci was performed with the Sanger sequencing method. MLST and *ftsI* typing was conducted following the instructions on the website PubMLST (https://pubmlst.org/ accessed on 20 December 2024).

The minimum spanning tree (MST) was generated on the online platform of PHYLOViZ Online (https://online.phyloviz.net/index accessed on 28 December 2024) [28]. MST is a graph-theoretic method that constructs evolutionary relationships based on gene sequence similarity. It reveals genetic associations and evolutionary pathways among bacterial populations through mathematical modeling. The data utilized in the MST analysis consisted of the bacterial allelic profiles obtained through the MLST analysis.

### 2.6. Detection of β-Lactam Resistance-Related Genes

The presence of the β-lactamases *bla*_TEM-1_ and *bla*_ROB-1_ was confirmed by PCR. The primers used in this study were provided by Yuan et al. [10] and are listed in the Supplementary Materials.

Sequencing of the gene *ftsI* [8] was performed with the Sanger sequencing method. for *ftsI* typing we followed the instructions on the website PubMLST (https://pubmlst.org/ accessed on 20 December 2024). The mutation patterns of the *ftsI* gene are divided into three groups according to specific amino acid substitutions [7,9]. Groups I and II contain the first-stage substitutions Arg517His and Asn526Lys, respectively. Group III and group III-like are defined by the second-stage Ser385Thr substitution in addition to the first-stage substitutions Arg517His and Asn526Lys. Group III+/III-like+ contains the third-stage Leu389Phe substitution, in addition to the substitutions observed in group III/III-like [7]. All *ftsI* alleles were grouped according to their mutation pattern.

### 2.7. Antibiotic Susceptibility Test (AST)

All the antibiotic sensitivity tests were conducted with the Etest method. The breakpoints applied in this study were derived from both *CLSI-M100^TM^ Performance standards for Antimicrobial Susceptibility Testing*, 34th Edition [29], and EUCAST breakpoint tables for interpretation of MICs and zone diameters, version 14.0 [30].

All the *H. influenzae* strains were cultured on chocolate agar plates (OXOID) for 16 h overnight. The inoculum suspension was prepared by picking several colonies with a cotton swab and suspending them in 0.85% NaCl saline to achieve a density equivalent to that of a 0.5 McFarland standard. The inoculum was evenly spread across over the entire surface of a Haemophilus test medium plate to which X factor (hematoxylin), V factor (NAD), and yeast extract were added based on MH medium. The plates were incubated in the presence of 5% CO_2_ at 35 °C for 18 h. The following antibiotics were tested using the Etest method: ampicillin (AMP), ampicillin/sulbactam (SAM), ceftriaxone (CRO), imipenem (IMP), meropenem (MEM), levofloxacin (LEV), tetracycline (TET), azithromycin (AZM), and trimethoprim–sulfamethoxazole (SXT) (all Etests were from Bio-KONT^®^, Wenzhou, China). The MIC values were measured and interpreted according to both CLSI and EUCAST breakpoints.

All isolates demonstrating resistance to imipenem (IMP) in the antimicrobial susceptibility testing (AST) results were subjected to the modified Hodge test (MHT) for the phenotypic confirmation of carbapenemase production [31].

MHT is a phenotypic assay recommended by the CLSI for screening carbapenemase production in Enterobacteriaceae. Its principle relies on the inactivation of carbapenem antibiotics by carbapenemases produced by the test strain, leading to enhanced growth of an indicator strain (*Escherichia coli* ATCC 25922) in the vicinity of the test strain’s inoculation line. Although *H. influenzae* belongs to the Pasteurellaceae family, if there are carbapenemases produced by *H. influenzae*, they could possibly also enhance the growth of *E. coli*. We tentatively employed this method to confirm the presence of the enzymes.

### 2.8. Analysis of the Amino Acid Sequences of the acrRAB Gene Cluster Coding for the Multi-Drug Efflux Pump and the Gene ompP2 of Porin

In most Gram-negative bacterial genomes, the genes for acrR, acrA, and acrB are present. AcrA functions as a multi-drug efflux pump membrane fusion lipoprotein, whereas AcrB is a multi-drug efflux pump resistance–nodulation–division (RND) permease [32]. AcrR acts as the regulatory gene of both acrA and acrB. These three genes are closely arranged and designated as acrRAB. The multi-drug efflux pump is designated as AcrAB. They could affect drug susceptibility [33]. Variations in the major outer membrane protein P2 (ompP2) could also affect the susceptibility of *H. influenzae* strains to imipenem [34]. To determine whether increased efflux of imipenem played a role in the isolates with higher imipenem MICs, the acrRAB coding sequence was determined and compared to that of *H. influenzae* Rd KW20. Additionally ompP2 from the strains with higher imipenem MICs was also sequenced. The nucleotide and amino acid sequences were analyzed using the wild-type strain Rd KW20 as a reference with the BLAST blastn (2.14.0+) and blastp (2.14.0+) suite pipeline and ESPript 3.0 [35]. The primer sequences used to amplify the genes were provided by Zwama M et al. [33] and Cherkaoui et al. [34] and are listed in the Supplementary Materials.

### 2.9. Statistical Analysis

The statistical software utilized in this study was SPSS (Version 11.0, IBM Corp., Armonk, NY, USA), and the statistical methods employed were the chi-square test and Wilson’s method for proportion confidence intervals.

## 3. Results

### 3.1. Relevant Data from the Enrolled Patients

From January 2024 to August 2024, there were 56 *H. influenzae* strains (14.5%) identified from 386 non-repeated nasopharyngeal tissue samples collected from AH patients who underwent endoscopic adenoidectomy in the Department of Otolaryngology.

The 386 AH patients ranged in age from 2 years old to 15 years old; 59 were 2–5 years old, 201 were 6–9 years old, and 126 patients were 10–15 years old. The group of 2–5 year-old patients had a significantly higher positive rate of *H. influenzae* presence compared to the group of 6–9 year-old patients (*p* = 0.0023) or the group of 10~15 year-old patients (*p* = 0.0001) (Table 1). The sex ratio of the patients was 228 males to 158 females.

### 3.2. Capsular Genotyping Results

We conducted the PCR test of the *bexA* and *bexB* genes from all the strains. Six strains exhibited a band of the target size of *bexA*, and none of them showed positive results for *bexB*. Then we performed the q-PCR tests for the serotypes from A to E for the *bexA*-positive strains. However, all the capsular genotyping tests yielded negative results. It is believed that the false positive results were due to the 5′ primer hybridized in the deleted region of *bexA* [24]. The capsular genotyping test results indicated that all the strains isolated in this study were un-typeable.

### 3.3. MLST Results, ftsI Allele Grouping Data, and Presence of the β-lactamases bla_TEM-1_ and bla_ROB-1_

Forty-nine of the fifty-six *H. influenzae* strains were successfully sub-cultured and numbered in our strain bank. All enrolled *H. influenzae* strains were re-identified using 16s rRNA sequencing. The 49 strains with their strain bank numbers are listed in Table 2. In total, 22 sequence types (STs) and 13 clonal complexes (CCs) were detected. There were 3 strains of CC395, 4 strains (8.1%) of CC155, 5 strains (10.2%) of CC57, 7 strains (14.3%) of CC3 and CC107, and 13 strains (26.5%) of CC11. Other CCs were present with only one or two strains. The dominant STs were ST103 (11 strains, 22.4%), ST57 (5 strains, 10.2%), and ST107 (5 strains, 10.2%). The most diverse clonal complex was CC3, which was composed of ST4, ST143, ST436, ST481, and ST 2757.

### 3.4. Minimum Spanning Tree

**Figure 1:**
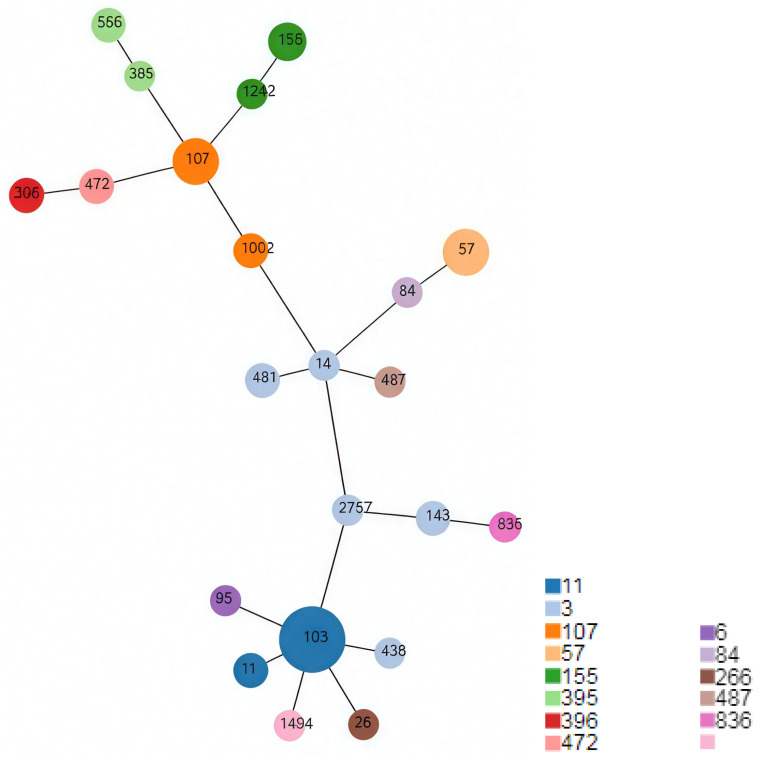
The minimum spanning tree for the 49 strains of *H. influenzae*.

The minimum spanning tree was drawn based on the MLST data of the 49 NTHi strains (Figure 1). It is evident that the ST 103 and ST107 are the two most prominent central nodes which look like the ancestors of several other STs. Additionally, ST14, ST481, ST2757, and ST143 are all closely related and belong to CC3, which is marked in light greyish blue. CC11, CC3, and CC107 are three main clonal complexes, each with various minority branches.

The colors in the figure correspond to different CCs. The size of each node represents the number of strains within each ST, with larger nodes indicating a higher number of strains. The pink color in the legend indicates that there is no clonal complex information available for ST1494.

### 3.5. Mutation Patterns of the ftsI Alleles

All *ftsI* alleles of the 49 strains, including mutation substitutions and the presence of *bla*_TEM-1_ and *bla*_ROB-1_, are listed in Table 3. The most frequent allele was 88, detected in 14 strains, while the second most frequent was allele 26, found in 6 strains. Alleles 88 and 26 both belong to the group III-like+.

There were 28 strains classified as *ftsI* group III-like+, 6 strains as group III-like, 8 strains as group III+, 2 strains as group IIa, 1 strain as group III+IIb, 1 strain as miscellaneous, and 3 strains as wild-type without important mutations. The PCR tests for the gene coding for *bla*_TEM-1_ showed a high positive rate of 95.9% (47/49). The detection rate for *bla*_ROB-1_ was significantly lower, being only of 18.36% (9/49). Eight strains harbored both *bla*_TEM-1_ and *bla*_ROB-1_ genes. Only one strain was positive for the *bla*_ROB-1_ gene alone. Another single strain tested negative for both *bla*_TEM-1_ and *bla*_ROB-1_ genes.

The substitution Asp-350 to Asn was detected in 93.9% (46/49) of the strains, including three wild-type strains, while Ser-357 to Asn was found in 71.4% (35/49) of the strains. The substitution Gly-490 to Glu was present in only four strains, all of which were in group III+. The substitutions Ala-502 to Val and Asn-526 to His were each detected in only one strain. The most frequent three amino acid substitutions were Met-377 to Ile, Ser-385 to Thr, and Leu-389 to Phe. This triplet mutation pattern was observed in 77.6% (38/49) of the strains, in addition to the substitutions of Arg-517 to His and Asn-526 to Lys (Table 3).

### 3.6. Summary of the AST Results

The MIC results were analyzed using two sets of break points (BPs) provided by the CLSI [29] and EUCAST [30]. The highest sensitivity was observed in the AST results for TET and being as high as 100% with both BP criteria, with a median MIC of 0.25 µg/mL. The lowest sensitivity was observed in the AST results for AMP and corresponded to 10.2% with both BP criteria, with a median MIC of ≥256 µg/mL. The AST results for MEM demonstrated 100% sensitivity with the EUCAST BPs, while the susceptibility to imipenem, judged based on the CLSI criteria, appeared concerningly low. CRO, MEM, TET, and LEV showed 100% sensitivity according to at least one set of BP criteria. AMP, SAM, AZM, and SXT showed sensitivity from 10.2% to 34.7% (Table 4).

There were 44 (89.8%) β-lactamase-positive strains with AMP resistance designated as β-lactamase-positive with AMP resistance (BLPAR). The only β-lactamase-free strain was sensitive to AMP (Supplementary Materials) and could not be designated as β-lactamase-negative with AMP resistance (BLANR).

The AST results for IMP were surprising. Even according to the less stringent criterion of EUCAST BPs, the sensitivity rate was only 91.8%. The AST results for all strains with an MIC for imipenem equal to or higher than 2 µg/mL, along with their strain bank numbers, are listed in Table 5. These strains were classified as imipenem-resistant according to both sets of BPs. They were all confirmed as carbapenemase-negative by MHT. It is notable that all of them had the Asn-526 substitution without the Arg517His substitution. The detailed AST results are presented in the Supplementary Materials.

### 3.7. The Amino Acid Sequence Pattern of acrRAB Gene Clusters and the Gene ompP2 Coding for Porin

**Figure 2:**
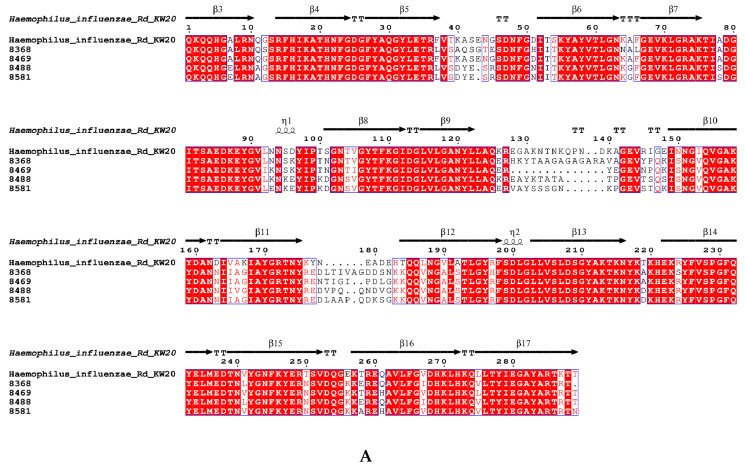

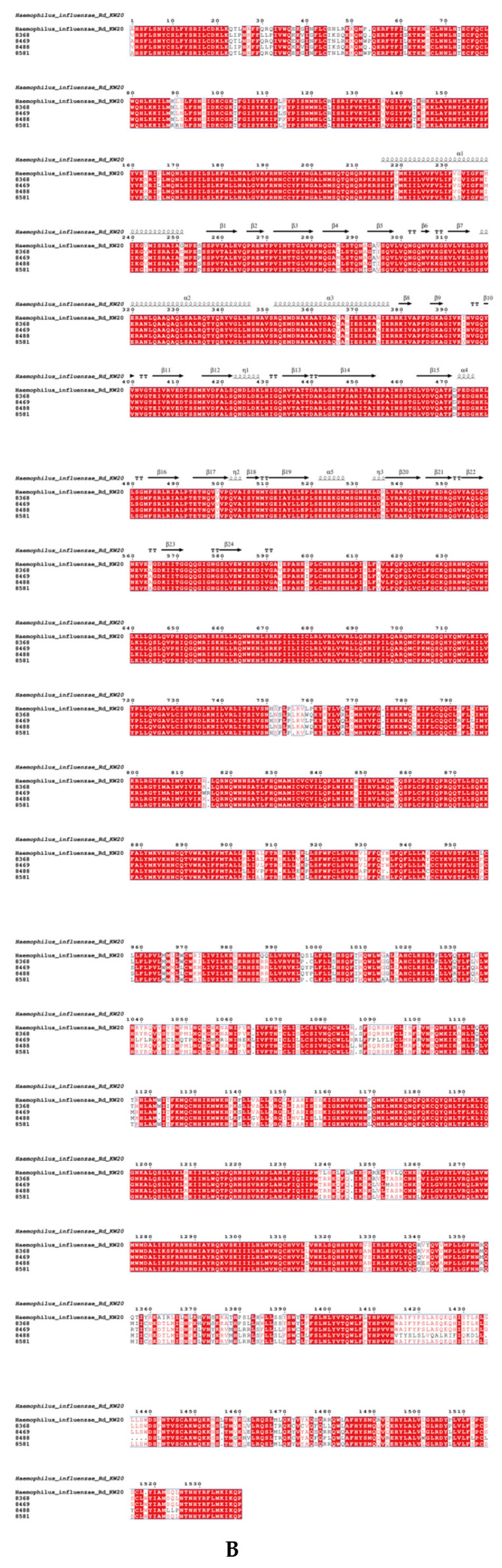
(**A**) The amino acid substitutions in *ompP2* in the 4 imipenem-resistant strains compared with the reference *H. influenzae* Rd KW20 strain; (**B**) the amino acid substitutions in protein expressed by *acrRAB* gene cluster in the 4 imipenem-resistant strains compared with the reference *H. influenzae* Rd KW20strain. The secondary structure of the amino acid sequences is labelled as “a1, β1, a2, β2…” to ensure clear identification of its structural elements. Coloring indicates sequence similarity. The threshold for global similarity score was set at 0.7. The in-group similarity score (ISc) is a classical similarity score to determine similarity within each group. The cross-group score (XSc) is the similarity score average for every sequence pair, with each sequence belonging to a different group. The total similarity score (TSc) is the mean of ISc and XSc. The colors were chosen according to the following rule: red box, white character means strict identity; red character means ISc higher than threshold of 0.7; blue frame: TSc higher than threshold of 0.7. Black character means lower similarity.

The amino acid sequence identity percentages of the *acrRAB* gene cluster between the four imipenem-resistance strains and the reference *H. influenzae* Rd KW20 strain ranged from 88.12% to 94.94%. Strains No. 8488, No. 8581, and No. 8368 exhibited identity percentages of 77.82%, 77.21%, and 77.10% for *ompP2,* respectively, whereas No. 8469 showed 82.94% identity (Table 6). Figure 2A presents the amino acid substitutions in porin in the four imipenem-resistant strains compared with the reference *H. influenzae* Rd KW20 strain, whereas the amino acid substitutions in AcrAB multi-drug efflux pump are shown in Figure 2B. All the point mutations in the DNA sequences of ompP2 and acrRAB gene cluster in the four imipenem-resistant strains compared with the reference *H. influenzae* Rd KW2 strain can be found in the Supplementary Materials.

Figure 2: The amino acid substitutions in *ompP2* and *acrRAB* gene cluster in the 4 imipenem-resistant strains compared with the reference *H. influenzae* Rd KW20 strain.

## 4. Discussion

A recent research conducted within a population similar to ours reported a similar positive rate of *H. influenzae* among pediatric AH patients [36]. We also observed that the younger the patients, the higher the positive rate of *H. influenzae*. For patients under 5 years of age, the positive rate could be as high as 30%. Our findings are similar to previous research findings [4,5], although the positive rate could be even higher if molecular methods were applied [5]. Since all capsule genotyping tests were negative, all *H. influenzae* strains in our study were identified as NTHi. NTHi is a ubiquitous commensal of the human upper respiratory tract. In most cases, it does not lead to disease. However, since the introduction of the Hib vaccine, the burden of *H. influenzae*-related infections has been increasingly dominated by NTHi. NTHi is able to exert differential binding to the host tissue through the use of a broad range of adhesins. NTHi’s survival and pathogenicity utilize several virulence factors including complement resistance, biofilm, and modified immunoglobulin responses [37]. For instance, outer membrane vesicles (OMVs) from NTHi have been shown to increase the secretion of interleukin (IL)-1β and IL-17, contributing to neutrophilic inflammation in asthma [38]. NTHi is also a known cause of meningitis and post-meningitis hearing loss in children, though antibiotic side effects may also contribute to hearing loss [39]. A Canadian multi-center study reported that NTHi accounted for 36% of *H. influenzae* bacteremia in children under 12 months [40]. These findings underscore the importance of NTHi as a commensal bacteria, particularly among young children, given its potential for causing invasive disease. The role played by NTHi in the development of AH among pediatric patients, especially those under 5 years, warrants further studies.

In our study, we identified three dominant clonal complexes (CCs) in *H. influenzae* carried by pediatric patients with adenoid hypertrophy. The most prevalent CC was CC11, accounting for over a quarter of the strains. CC3 and CC107 were the second and third most frequently identified CCs, respectively. On the minimum spanning tree these three CCs were also the most prominent, representing the three main ancestors of other minor CCs. Previous research in southern China reported that CC107, CC3, and CC487 were the dominant clonal complexes in pediatric patients [41]. Our finding that CC3 was the most genetically diverse clonal complex is consistent with that report [41]. Studies in Beijing and nearby regions showed more varied distributions of STs and CCs, with no overlap in dominant STs when compared to our study [42]. A study in Shanghai also demonstrated a higher level of diversity, although ST107 was also the most frequently detected ST, similar to our findings [43]. Our study is the first to report CC11 as the dominating MLST type of *H. influenzae* among children.

The lower genetic diversity observed in our data may be attributable to the relatively homogeneous population of patients in our study. All patients lived in nearby communities and suffered from adenoid hypertrophy, suggesting that ST103 from CC11 could reflect the predominant strains colonizing the adenoids of this population. Our hospital, located in a densely populated community complex in Beijing, houses around 500,000 people, which may explain the localized predominance of these clonal complexes.

High carriage rates of NTHi in both healthy children and those with adenoid hypertrophy have been briefly reported in China in 2022 [44]. In that study, β-lactamase-producing strains accounted for 44.7% of the isolates; however, our data showed a strikingly high positivity rate for the β-lactamase genes, with 18.36% of the strains carrying two different β-lactamases. Such high positivity rates for β-lactamase genes, especially the *bla*_ROB-1_ gene, were rarely reported in Chinese studies [41,42,43].

In addition, we found that 93.9% of the strains harbored mutations in the *ftsI* gene, which encodes PBP3. This rate is higher than the 80% alteration rate reported by Zhou et al. [41]. The majority of the strains in our study belonged to group III-like+, whereas group I strains were scarce, which is consistent with other research studies in Eastern Asia [7,41,42,43]. The high prevalence of PBP3 mutations in local NTHi strains is concerning. Studies from Korea suggested that group III-like+ strains exhibited the highest ceftriaxone (CRO) MICs [7], while our data indicated that Asn-526 substitutions may be associated with imipenem resistance.

The low susceptibility to ampicillin (10.2%) is consistent with the high rates of β-lactamase genes and PBP3 mutations. Besides the only β-lactamase-free strain, No. 8654, four strains were ampicillin-sensitive, despite carrying both β-lactamase genes and PBP3 mutations, likely due to phenotypic and genotypic discrepancies. Previous research has suggested that recombination events involving horizontal gene transfer could result in diverse MICs for strains with identical *ftsI* genes [34,45]. The high susceptibility rates for tetracycline (TET) and levofloxacin (LEV) are consistent with findings in other studies from China [41,42,43], likely due to the limited use of these antibiotics in pediatric patients.

Among the four β-lactamase-positive ampicillin-resistant (BLPAR) strains that exhibited imipenem resistance, none were carbapenemase-positive. This suggests that the resistance mechanism may be related to efflux pumps and outer membrane porin proteins [33,34]. The amino acid sequences of the *acrRAB* gene cluster from these strains were nearly identical to those from the wild-type *H. influenzae* Rd KW20 strain, while the *ompP2* sequences exhibited significant variability. A reasonable speculation is that porin plays a more important role than the AcrAB multi-drug efflux pump in the mechanisms of carbapenemase-free imipenem resistance. It is possible that the altered peptide sequences of porin in these strains cause reduced permeability, leading to increased imipenem resistance. Further research on mutations in porin is necessary to clarify their role in β-lactam resistance. This finding may assist clinical guidelines in reevaluating the role of imipenem in the treatment of *H. influenzae*. This study has several limitations, including the drawbacks of being a single-center study, the failure to detect rare β-lactamase types such as *bla*_ROB-11_ and *bla*_TEM-15_, and the lack of consideration for the impact of the season on the detection rate of *H. influenzae*.

In conclusion, the BLPAR NTHi of CC11 appeared as a significant epidemic clonal complex among children with adenoid hypertrophy. Pediatricians and researchers should be aware of the high frequency of β-lactamase genes and the resistance to imipenem of *H. influenzae* carried in the adenoids of young children in Beijing. Further studies are also needed to elucidate the role played by *H. influenzae* in the development of adenoid hypertrophy among patients under 5 years.

## Figures and Tables

**Table 1:** The number of *H. influenzae* strains isolated from AH patients in different age groups.

Age Group (Number of AH Patients)	Number of Strains	Ratio (95% Confidence Interval)	Chi-Square Test (Trend)		
2–5 years old (59)	18	30.51% (20.33–43.06%)	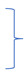	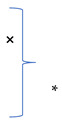	*p* = 0.0003
6–9 years old (201)	27	13.43% (9.46–18.78%)			
10–15 years old (126)	11	8.73% (4.99–14.85%)			

× *p* = 0.0023 * *p* = 0.0001.

**Table 2:** The numbers of all the STs and CCs.

CC	ST	Number
3	14	1
	143	2
	436	1
	481	2
	2757	1
6	95	1
11	103	11
	11	2
57	57	5
84	84	1
107	107	5
	1002	2
155	155	3
	1242	1
266	266	1
395	395	1
	556	2
396	396	2
472	472	2
487	487	1
836	836	1
-	1494	1

-: No available data for the typing of CCs.

**Table 3:** The mutation patterns of the *ftsI* alleles and the presence of the β-lactamases *bla*_TEM-1_ and *bla*_ROB-1_.

*ftsI* Allele	*ftsI* Group	Number of Strains	Position 350	Position 357	Position 377	Position 385	Position 389	Position 490	Position 502	Position 517	Position 526	*bla*_TEM-1_	*bla*_ROB-1_
22	III+	2	Asp350Asn	Ser357Asn	Met377Ile	Ser385Thr	Leu389Phe	Gly490Glu			Asn526Lys	+	-
26	III-like+	4	Asp350Asn		Met377Ile	Ser385Thr	Leu389Phe			Arg517His		+	-
		2										+	+
33	III-like	2	Asp350Asn	Ser357Asn	Met377Ile	Ser385Thr				Arg517His		+	+
		1										+	-
		1										-	-
40	III+	2	Asp350Asn	Ser357Asn	Met377Ile	Ser385Thr	Leu389Phe				Asn526Lys	+	-
		1										-	+
88	III-like+	13	Asp350Asn	Ser357Asn	Met377Ile	Ser385Thr	Leu389Phe			Arg517His		+	-
		1										+	+
98	IIa	1									Asn526Lys	+	-
107	III-like+	2	Asp350Asn	Ser357Asn	Met377Ile	Ser385Thr	Leu389Phe			Arg517His		+	+
		1										+	-
142	IIa	1									Asn526Lys	+	-
185	III+	2	Asp350Asn	Ser357Asn	Met377Ile	Ser385Thr	Leu389Phe	Gly490Glu			Asn526Lys	+	-
194	III-like+	3	Asp350Asn	Ser357Asn	Met377Ile	Ser385Thr	Leu389Phe			Arg517His		+	-
197	Miscellaneous	1	Asp350Asn		Met377Ile	Ser385Thr	Leu389Phe				Asn526His	+	-
200	III+	1	Asp350Asn	Ser357Asn	Met377Ile	Ser385Thr	Leu389Phe				Asn526Lys	+	-
202	III-like+	1	Asp350Asn	Ser357Asn	Met377Ile	Ser385Thr	Leu389Phe			Arg517His		+	-
275	WT	1	Asp350Asn									+	-
331	III-like	1	Asp350Asn	Ser357Asn		Ser385Thr				Arg517His		+	+
		1										+	-
336	WT	2	Asp350Asn									+	-
370	III-like+	1			Met377Ile	Ser385Thr	Leu389Phe			Arg517His		+	-
374	III+IIb	1	Asp350Asn	Ser357Asn	Met377Ile	Ser385Thr	Leu389Phe		Ala502Val		Asn526Lys	+	-

WT: wild-type.

**Table 4:** The summary of the AST results for 49 strains of *H. influenzae*.

Antibiotic	Susceptibility by CLSI BPs	Susceptibility by EUCAST BPs	Mode of MIC (µg/mL)	Median and Range of MIC (µg/mL)
MEM	95.9%	100%	0.38	0.25 (from 0.032 to 1.5)
CRO	100%	59.2%	0.048	0.094 (from 0.008 to 1)
IMP	42.9%	91.8%	0.5	0.75 (from 0.048 to ≥32)
AMP	10.2%	10.2%	≥256	≥256 (from 0.5 to ≥256)
SAM	28.6%	14.3%	4	4 (from 0.25 to ≥32)
LEV	100%	98.0%	0.032	0.032 (from 0.008 to 1)
TET	100%	100%	0.25	0.25 (from 0.064 to 0.25)
SXT	32.7%	32.7%	≥32	1 (from 0.016 to ≥32)
AZM	34.7%	*	≥256	64 (from 0.5 to ≥256)

BPs: break points; * no BP was provided.

**Table 5:** The features of 4 imipenem-resistant *H. influenzae* strains.

Bank Number	MEM	CRO	IMP	AMP	SAM	LEV	TET	SXT	AZM	*ftsI* Group	*bla*_TEM-1_	*bla*_ROB-1_
8488	0.38	0.024	4	256	4	0.032	0.25	32	256	IIa	+	−
8469	1	0.125	8	256	4	0.032	0.25	32	64	III+	+	−
8581	0.38	0.19	≥32	256	16	0.032	0.25	16	256	III+	+	−
8368	0.25	1	4	256	4	1	0.25	32	256	Miscellaneous	+	−

**Table 6:** Similarity in nucleic acid sequences and amino acid sequences for the *acrRAB* gene cluster and *ompP2* between the studied strains and the Rd KW2 reference strain.

Bank Number	*acrRAB* Gene Cluster and AcrAB Multi-Drug Efflux Pump		*ompP2* and Porin	
	Average Identity (%) for Nucleotides	Average Identity (%) for Amino Acids	Average Identity (%) for Nucleotides	Average Identity (%) for Amino Acids
8368	97.41	93.77	83.33	77.10
8469	96.72	92.10	86.85	82.94
8488	95.20	88.12	84.47	77.82
8581	97.85	94.94	85.20	77.21

## Data Availability

The original contributions presented in this study are included in the article/Supplementary Materials. Further inquiries can be directed to the corresponding authors.

## Supplementary Materials

The following supporting information can be downloaded at: https://www.mdpi.com/article/10.3390/microorganisms13081764/s1, Figure S1: Modified Hodge Test result of No.8581; Figure S2: Point mutations of DNA sequences of *ompP2* (A) and *acrRAB* (B) gene cluster of the 4 imipenem-resistant strains comparing with the reference *H. influenzae* Rd KW20; Table S1: Primers used in the study; Table S2: The primers and probes used to do the serotyping of *H.influenzae*; Table S3: The detailed AST results of MEM, CRO, IMP, AMP and SAM; Table S4: The detailed AST results of LEV, TET, SXT and AZM.
