# Supplementary material for: Molecular and Drug Resistance Characteristics of Haemophilus influenzae Carried by Pediatric Patients with Adenoid Hypertrophy
Source: Microorganisms. 2025 Jul 29;13(8):1764. doi: 10.3390/microorganisms13081764 (PMC12388765; doi:10.3390/microorganisms13081764)
Supplement: Supplementary file 1 [file microorganisms-13-01764-s001.zip › microorganisms-3745136-supplementary.pdf]

Supplementary Material

Table S1. Primers used in the study

Locus	Forward	Reverse	Length	Gene product	Reference
adk	5'-GGTGCACCGGGTGCAGGTAA-3'	5'-CCTAAGATTTTATCTAACTC-3'	477	Adenylate kinase	Meats E, Feil EJ, Stringer S, et al. Characterization of encapsulated and noncapsulated Haemophilus influenzae and determination of phylogenetic relationships by multilocus sequence typing. J Clin Microbiol. 2003;41(4):1623-1636.
atpG	5'-ATGGCAGGTGCAAAAGAGAT-3'	5'-TTGTACAACAGGCTTTTGCG-3'	447	ATP synthase F1 subunit gamma	
frdB	5'-CTTATCGTTGGTCTTGCCGT-3'	5'-TTGGCACTTTCCACTTTTCC-3'	489	Fumarate reductase iron-sulfur protein	
fucK	5'-ACCACTTTCGGCGTGGATGG-3'	5'-AAGATTTCCCAGGTGCCAGA-3'	345	Fuculokinase	
mdh	5'-TCATTGTATGATATTGCCCC-3'	5'-ACTTCTGTACCTGCATTTTG-3'	405	Malate dehydrogenase	
pgi	5'-GGTGAAAAAATCAATCGTAC-3	5'-ATTGAAAGACCAATAGCTGA-3'	468	Glucose-6-phosphate isomerase	
recA	5'-ATGGCAACTCAAGAAGAAAA-3'	5'-TTACCAAACATCACGCCTAT-3'	426	RecA protein	
ftsI	5'-CCTTTCGTTGTTTTAACCGCA-3'	5'-AGCTGCTTCAGCATCTTG-3'	770	PBP3	Skaare D, Allum AG, Anthonisen IL, et al. Mutant ftsI genes in the emergence of penicillin-binding protein-mediated beta-lactam resistance in Haemophilus influenzae in Norway. *Clin*

					Microbiol Infect. 2010;16(8):1117-1124.
TEM-1	5'-TAAGAGAATTATGCAGTGCTGCC-3'	5' -TCCATAGTTGCCTGACTCCC-3'	458		Yuan M, Ma M, Jiang H, et al. Characterization of Serotypes and Molecular Drug Resistance Patterns of Haemophilus influenzae in Kunming Children. Pol J Microbiol. 2023;72(2):125-131.
ROB-1	5'-AAGCCCGCATTGGCGTTTAT-3'	5'-GGGCAATCGTCATGCCTTTG-3'	521		
bexA	5'-CGTTTGTATGATGTTGATCCAGAC-3'	5'-TGTCCATGTCTTCAAAATGATG-3'	343		
bexB	bexB.1F GGTGATTAACGCGTTGCTTATGCG	bexB.1R TTGTGCCTGTGCTGGAAGGTTATG	567		Davis GS, Sandstedt SA, Patel M, Marrs CF, Gilsdorf JR. Use of bexB to detect the capsule locus in Haemophilus influenzae. J Clin Microbiol. 2011 Jul;49(7):2594-601. doi: 10.1128/JCM.02509-10.
	bexB.FLF TCATTGTGGCTCAACTCCTTTACT	bexB.FLR AGCTATTCAAGGACGGGTGATTAACGC	760		
acrRAB	5'-GCTCATTCATTTTTGAGCAATTATTGCTCCTTATTT-3'	5'-TTAATGGTGATGGTGATGGTGGGTTGTTTTATTTT-3'	831	AcrAB-TolC efflux pump	Zwama M, Yamaguchi A, Nishino K. Phylogenetic and functional characterisation of the Haemophilus influenzae multidrug efflux pump AcrB. Commun Biol. 2019;2:340.

					Published 2019 Sep 13. doi:10.1038/s42003-019-0564-6
ompP2	5'-ATAACAACGAAGGGACTAACG-3'	5'-ACCTACACCCACTGATTTTTC-3'	~1000	porin (ompP2)	Cherkaoui A, Diene SM, Renzoni A, Emonet S, Renzi G, Francois P, Schrenzel J: Imipenem heteroresistance in nontypeable Haemophilus influenzae is linked to a combination of altered PBP3, slow drug influx and direct efflux regulation. Clinical microbiology and infection : the official publication of the European Society of Clinical Microbiology and Infectious Diseases 2017, 23(2):118 e119-118 e119. 1992;6(15):2107-2112.

Table S2. The primers and probes used to do the serotyping of *H.influenzae*

Locus	Forward	Reverse	Length	Fluorophore of the probe
Hi-a(acsB)	GGTCTGCGGTGTCCTGTGT	CCGGTCATCTTTTATGCTCCAA	166	
Pb375i	TAATTTTCTTGC"T"CAATACCGCCTTCCCA			5'FAM, BHQ1 on "T", 3'SpC3
Hi-b(bcsB)	TGATGCATTGAAAGAAGGTGTAATTT	CCTGCGGTAATAACATGATCATAAA	167	
Pb244i	TGTCGTGCAG"T"AGCAAACCGTAACCTTACTC			5'FAM, BHQ1 on "T", 3'SpC3
Hi-c(ccsD)	CATTGGTGATGGTTCAGTTATTGG	TACAGCATTCAGCAATAATGGG	117	
Pb7726i	ATTGCA"T"CGCCGCAGGAGTTCCCG			5'FAM, BHQ1 on "T", 3'SpC3
Hi-d(dcsE)	CCTAAAATACGGACCTAGTGCTGCAC	CCGATGAGACCAAGTATGGTTA	44	
Pb2221i	AACGAGC"T"AGAGCTGGTGCTGAA			5'FAM, BHQ1 on "T", 3'SpC3
Hi-e(ecsH)	ACTAAAATATGGCCCAAACCCAC	CCGATGAGCCCAAGTATGATGA	66	
Pb1555i	AACGAGCAAAAGCCGG"T"GCGGAT			5'FAM, BHQ1 on "T", 3'SpC3
Hi-f(bexD)	CCCTGAAAGCGTTGACTTTG	CCAACTTCAGGACCAAGTCATTC	149	
Pb7242i	TGCTGCTAAC"T"CAGATGCATCAGCTCCTT			5'FAM, BHQ1 on "T", 3'SpC3
hpd	GGTTAAATATGCCGATGGTGTTG	TGCATCTTTACGCACGGTGTA	130	
Pb896i	TTGTGTACACTCCGT"T"GGTAAAAGAACTTGCAC			5'FAM, BHQ1 on "T", 3'SpC3

From https://iris.who.int/handle/10665/70765

Table S3. The detailed AST results of MEM, CRO, IMP, AMP and SAM

Number	MEM	CLSI BP(≤0.5ug/mL)	EUCAST BP(≤2ug/mL)	CRO	CLSI BP(≤2ug/mL)	EUCAST BP(≤0.125ug/mL)	IMP	CLSI BP(≤0.5ug/mL)	EUCAST BP(≤2ug/mL)	AMP	CLSI BP(≤1, 2, ≥4ug/mL)	EUCAST BP(≤1ug/mL)	SAM	CLSI BP(≤2/1, ≥4/2ug/mL)	EUCAST BP(≤1ug/mL)

8342	0.064	S	S	0.016	S	S	0.125	S	S	0.5	S	S	0.25	S	S
8349	0.38	S	S	0.38	S	R	1	NS	S	256	R	R	8	R	R
8391	0.064	S	S	0.016	S	S	0.125	S	S	0.5	S	S	0.25	S	S
8409	0.38	S	S	0.38	S	R	1	NS	S	256	R	R	32	R	R
8410	0.38	S	S	0.125	S	S	0.5	S	S	256	R	R	8	R	R
8413	0.032	S	S	0.5	S	R	1	NS	S	32	R	R	16	R	R
8416	0.38	S	S	0.048	S	S	0.5	S	S	256	R	R	2	S	R
8489	0.064	S	S	0.25	S	R	1	NS	S	256	R	R	16	R	R
8494	0.19	S	S	0.094	S	S	1.5	NS	S	256	R	R	4	R	R
8495	0.38	S	S	0.125	S	S	0.5	S	S	256	R	R	8	R	R
8499	0.38	S	S	0.25	S	R	1	NS	S	256	R	R	16	R	R
8502	0.032	S	S	0.5	S	R	1	NS	S	32	R	R	16	R	R
8528	0.38	S	S	0.048	S	S	0.5	S	S	256	R	R	2	S	R
8535	0.032	S	S	0.5	S	R	1	NS	S	32	R	R	16	R	R
8544	0.38	S	S	0.048	S	S	0.5	S	S	256	R	R	2	S	R
8580	0.19	S	S	0.048	S	S	1.5	NS	S	256	R	R	4	R	R
8582	0.125	S	S	0.048	S	S	1.5	NS	S	256	R	R	4	R	R
8585	0.064	S	S	0.016	S	S	0.125	S	S	0.5	S	S	0.25	S	S
8586	0.19	S	S	0.048	S	S	2	NS	S	256	R	R	4	R	R
8602	0.38	S	S	0.125	S	S	0.5	S	S	256	R	R	8	R	R
8609	0.032	S	S	0.5	S	R	1	NS	S	32	R	R	16	R	R
8617	0.38	S	S	0.048	S	S	0.5	S	S	256	R	R	2	S	R
8623	0.38	S	S	0.25	S	R	1	NS	S	256	R	R	16	R	R
8625	0.064	S	S	0.016	S	S	0.125	S	S	0.5	S	S	0.25	S	S
8631	0.125	S	S	0.048	S	S	1.5	NS	S	256	R	R	4	R	R
8638	0.38	S	S	0.125	S	S	0.5	S	S	256	R	R	8	R	R

8639	0.19	S	S	0.048	S	S	0.75	NS	S	256	R	R	16	R	R
8644	0.032	S	S	0.5	S	R	1	NS	S	32	R	R	16	R	R
8654	0.125	S	S	0.048	S	S	2	NS	S	1	S	S	1	S	S
8659	0.38	S	S	0.048	S	S	0.5	S	S	256	R	R	2	S	R
8662	0.38	S	S	0.25	S	R	1	NS	S	256	R	R	16	R	R
8675	0.19	S	S	0.094	S	S	1.5	NS	S	256	R	R	4	R	R
8691	0.38	S	S	0.38	S	R	1	NS	S	256	R	R	32	R	R
8692	0.38	S	S	0.048	S	S	0.5	S	S	256	R	R	2	S	R
8356	0.125	S	S	0.048	S	S	2	NS	S	256	R	R	4	R	R
8488	0.38	S	S	0.024	S	S	4	NS	R	256	R	R	4	R	R
8381	0.048	S	S	0.064	S	S	0.38	S	S	2	I	R	4	R	R
8360	1.5	NS	S	0.5	S	R	0.75	NS	S	4	R	R	4	R	R
8367	0.5	S	S	0.5	S	R	0.5	S	S	8	R	R	4	R	R
8469	1	NS	S	0.125	S	S	8	NS	R	256	R	R	4	R	R
8507	0.032	S	S	0.064	S	S	0.048	S	S	2	I	R	8	R	R
8534	0.38	S	S	0.5	S	R	0.064	S	S	256	R	R	4	R	R
8581	0.38	S	S	0.19	S	R	32	NS	R	256	R	R	16	R	R
8594	0.25	S	S	0.125	S	S	0.75	NS	S	256	R	R	4	R	R
8606	0.5	S	S	0.19	S	R	0.75	NS	S	256	R	R	4	R	R
8368	0.25	S	S	1	S	R	4	NS	R	256	R	R	4	R	R
8591	0.094	S	S	0.008	S	S	0.5	S	S	64	R	R	2	S	R
8624	0.19	S	S	0.03	S	S	0.5	S	S	16	R	R	1	S	S
8640	0.064	S	S	0.019	S	S	0.5	S	S	32	R	R	1	S	S
S%		95.9%	100%		100%	59.2%		42.9%	91.8%		10.2%	10.2%		28.6%	14.3%

Table S4. The detailed AST results of LEV, TET, SXT and AZM

Number	LEV	CLSI BP(≤2ug/mL)	EUCAST BP(≤0.06ug/mL)	TET	CLSI BP(≤2, 4, ≥8ug/mL)	EUCAST BP(≤2ug/mL)	SXT	CLSI BP(≤0.5/9.5, 1/19-2/38, ≥4/76ug/mL)	EUCAST BP(≤0.5, ≥1ug/mL)	AZM	CLSI BP(≤4ug/mL), No BPs offered by EUCAST
8342	0.032	S	S	0.125	S	S	1	I	R	4	S
8349	0.016	S	S	0.064	S	S	0.016	S	S	256	NS
8391	0.032	S	S	0.125	S	S	1	I	R	4	S
8409	0.032	S	S	0.125	S	S	8	R	R	16	NS
8410	0.016	S	S	0.125	S	S	0.016	S	S	2	S
8413	0.032	S	S	0.25	S	S	32	R	R	256	NS
8416	0.016	S	S	0.125	S	S	1	I	R	64	NS
8489	0.032	S	S	0.25	S	S	32	R	R	256	NS
8494	0.032	S	S	0.25	S	S	0.032	S	S	256	NS
8495	0.016	S	S	0.125	S	S	0.016	S	S	2	S
8499	0.032	S	S	0.25	S	S	32	R	R	256	NS
8502	0.032	S	S	0.25	S	S	32	R	R	256	NS
8528	0.016	S	S	0.125	S	S	1	I	R	64	NS
8535	0.032	S	S	0.25	S	S	32	R	R	256	NS
8544	0.016	S	S	0.125	S	S	1	I	R	64	NS
8580	0.032	S	S	0.25	S	S	0.5	S	S	256	NS
8582	0.032	S	S	0.125	S	S	2	I	R	4	S
8585	0.032	S	S	0.125	S	S	1	I	R	4	S
8586	0.032	S	S	0.25	S	S	0.5	S	S	256	NS

8602	0.016	S	S	0.125	S	S	0.016	S	S	2	S
8609	0.032	S	S	0.25	S	S	32	R	R	256	NS
8617	0.016	S	S	0.125	S	S	1	I	R	64	NS
8623	0.032	S	S	0.25	S	S	32	R	R	256	NS
8625	0.032	S	S	0.125	S	S	1	I	R	4	S
8631	0.032	S	S	0.125	S	S	2	I	R	4	S
8638	0.016	S	S	0.125	S	S	0.016	S	S	2	S
8639	0.032	S	S	0.25	S	S	0.5	S	S	256	NS
8644	0.032	S	S	0.25	S	S	32	R	R	256	NS
8654	0.016	S	S	0.125	S	S	1	I	R	64	NS
8659	0.016	S	S	0.125	S	S	1	I	R	64	NS
8662	0.032	S	S	0.25	S	S	32	R	R	256	NS
8675	0.032	S	S	0.25	S	S	0.032	S	S	256	NS
8691	0.032	S	S	0.125	S	S	8	R	R	16	NS
8692	0.016	S	S	0.125	S	S	1	I	R	64	NS
8356	0.016	S	S	0.25	S	S	8	R	R	256	NS
8488	0.032	S	S	0.25	S	S	32	R	R	256	NS
8381	0.016	S	S	0.125	S	S	32	R	R	1	S
8360	0.016	S	S	0.25	S	S	0.032	S	S	2	S
8367	0.032	S	S	0.25	S	S	0.032	S	S	4	S
8469	0.032	S	S	0.25	S	S	32	R	R	64	NS
8507	0.008	S	S	0.064	S	S	0.016	S	S	0.5	S
8534	0.032	S	S	0.25	S	S	0.064	S	S	4	S
8581	0.032	S	S	0.25	S	S	16	R	R	256	NS
8594	0.016	S	S	0.25	S	S	32	R	R	256	NS
8606	0.032	S	S	0.25	S	S	16	R	R	128	NS

8368	1	S	R	0.25	S	S	32	R	R	256	NS
8591	0.032	S	S	0.25	S	S	0.064	S	S	2	S
8624	0.032	S	S	0.25	S	S	0.125	S	S	2	S
8640	0.032	S	S	0.25	S	S	32	R	R	64	NS
S%		100%	98.0%		100%	100%		32.7%	32.7%		34.7%

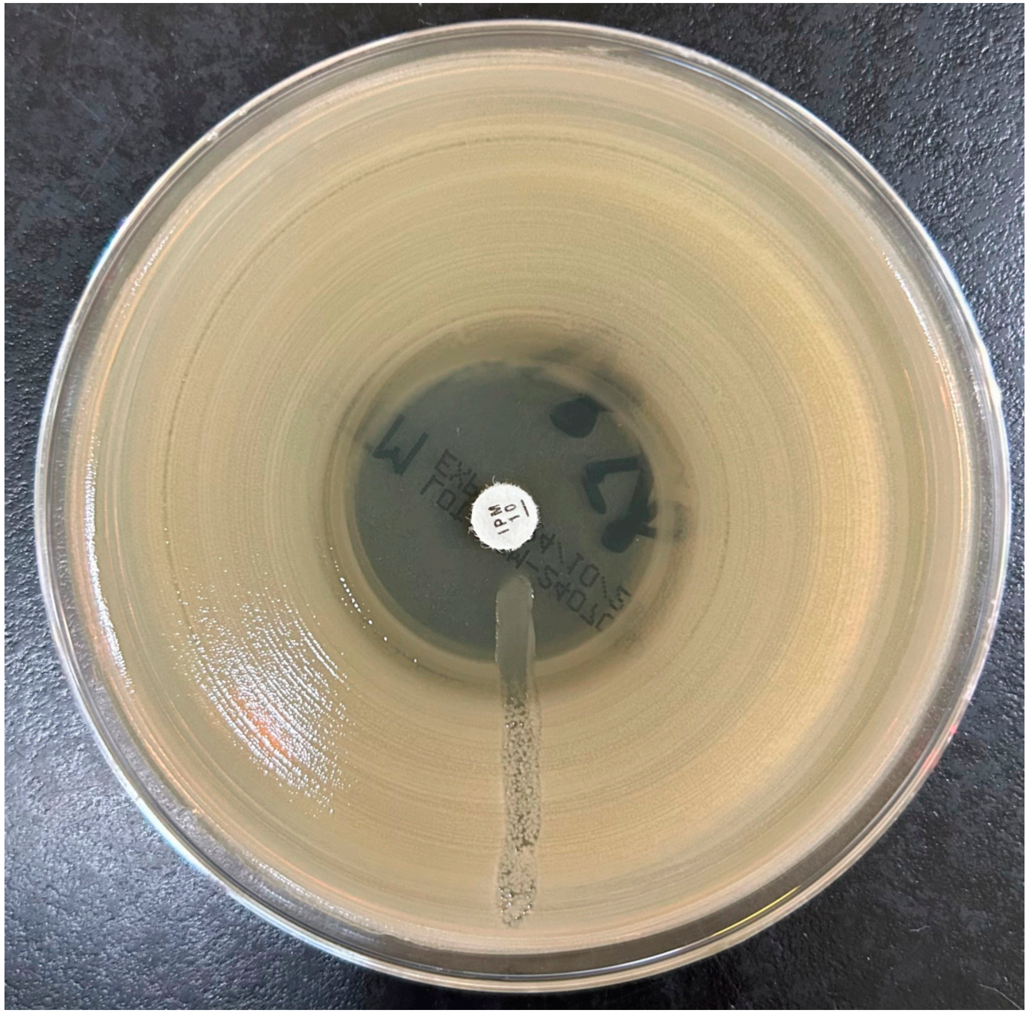

Figure S1. Modified Hodge Test result of No.8581

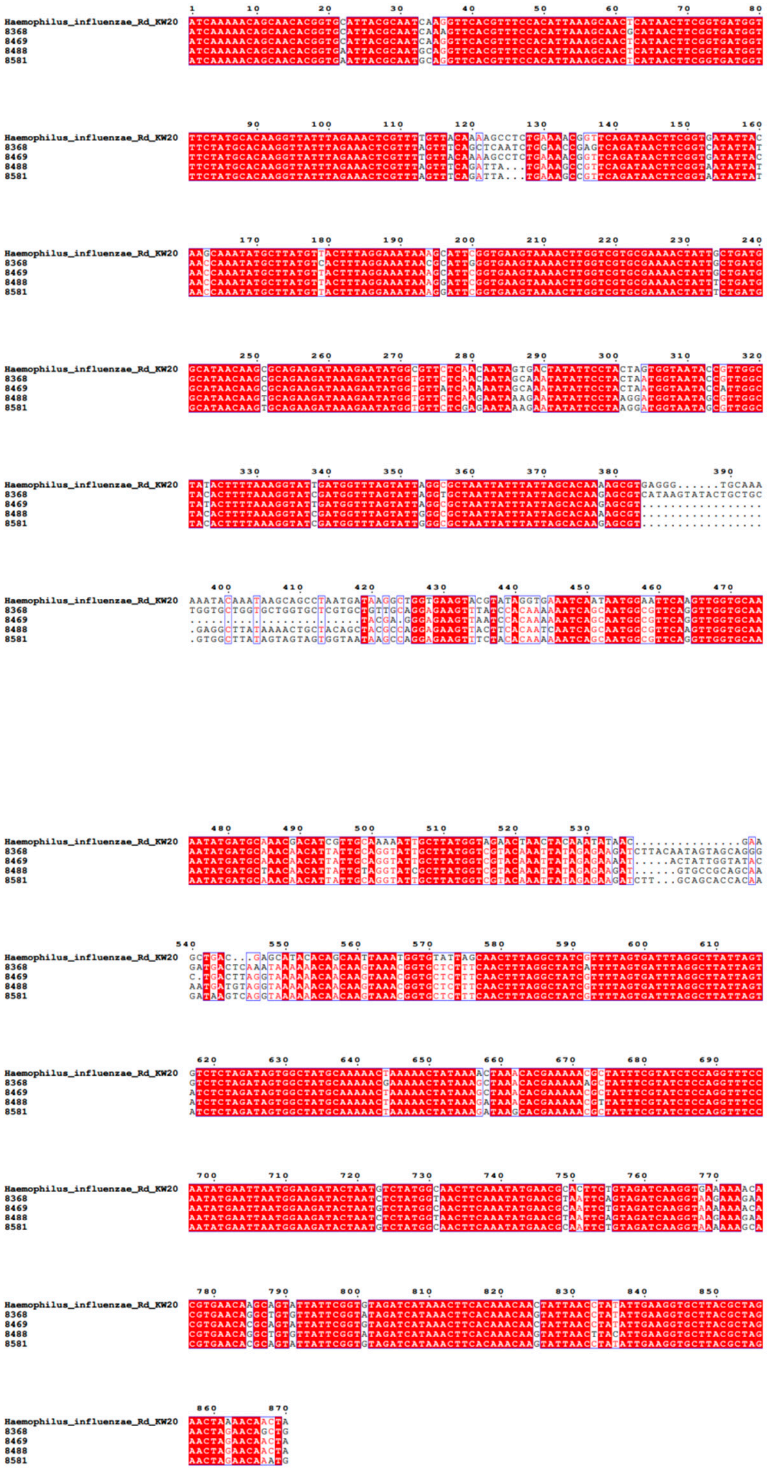

A

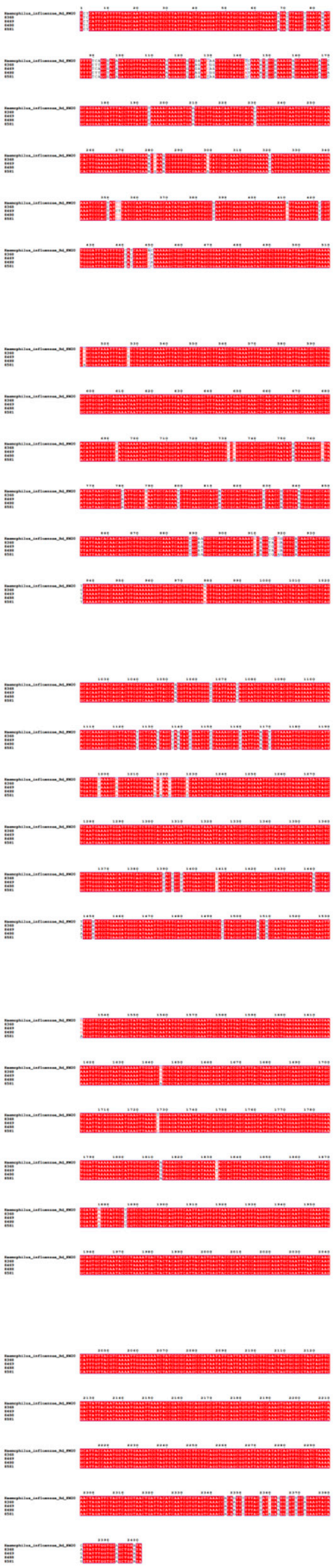

B

Figure S2. Point mutations of DNA sequences of *ompP2* (A) and *acrRAB* (B) gene cluster of the 4 imipenem-resistant strains comparing with the reference *H. influenzae* Rd KW20
